# Systematic Screening for SARS-CoV-2 to Detect Asymptomatic Infections: An Epitome of Taiwan's Outbreak

**DOI:** 10.1155/2022/6441339

**Published:** 2022-02-08

**Authors:** Chih-Chien Cheng, Chia-Chen Liu, Ju-Chuan Yen, Ting-Fang Chiu, Yi-Ning Liu, Thomas Y. Hsueh, Sheng-Huang Hsiao

**Affiliations:** ^1^Department of Education and Research, Taipei City Hospital, Taipei, Taiwan; ^2^School of Medicine, College of Medicine, Fu Jen Catholic University, New Taipei City, Taiwan; ^3^Department of Obstetrics and Gynecology, Taipei City Hospital, Zhongxiao Branch, Taipei, Taiwan; ^4^Taipei City University, Taipei, Taiwan; ^5^Graduate Institute of Biomedical Informatics, College of Medical Science and Technology, Taipei Medical University, Taipei, Taiwan; ^6^Department of Ophthalmology, Taipei City Hospital, RenAi Branch, Taipei, Taiwan; ^7^Department of Pediatrics, Taipei City Hospital, Taipei, Taiwan; ^8^Division of Urology, Department of Surgery, Taipei City Hospital, RenAi Branch, Taipei, Taiwan; ^9^Department of Urology, School of Medicine, National Yang Ming Chiao Tung University, Taipei, Taiwan; ^10^Department of Neurosurgery, Taipei City Hospital, RenAi Branch, Taipei, Taiwan; ^11^National Chenchi University, Taipei, Taiwan

## Abstract

**Background:**

Increased studies have revealed that asymptomatic carriers substantially impact the epidemic and that asymptomatic transmission is very common. Therefore, the asymptomatic transmission threat to the spread of the pandemic should not be neglected.

**Methods:**

The local outbreak in Taiwan, especially in Taipei City, is unprecedented and paramount and has claimed hundreds of lives, tens of thousands of cases, and enormous economic costs. As care providers and gatekeepers of infectious diseases, Taipei City Hospital has to perform regular polymerase chain reaction (PCR) results of admitted patients and healthcare workers (HCWs) to achieve these goals.

**Results:**

In this study, the results revealed a low positive rate of less than 1%, but the asymptomatic proportions could range from 42% to 46%, which bolsters that systematic screening was effective in controlling coronavirus disease-19 (COVID-19) of Novel Coronavirus or Severe Acute Respiratory Syndrome Coronavirus (SARS-CoV-2) and might be an exemplar to other similar scenarios. Universal screening of admitted patients may be important and necessary, especially in asymptomatic patients.

**Conclusions:**

Regular screening for healthcare providers is also important during this pandemic, and it is recommended that admitted patients and healthcare providers undergo systemic PCR testing.

## 1. Introduction

The coronavirus disease 2019 (COVID-19) outbreak has been an unprecedented event from Wuhan City in Hubei Province, China, since late December 2019. The World Health Organization (WHO) declared this outbreak a Public Health Emergency of International Concern (PHEIC) on January 30, 2020, and announced the COVID-19 pandemic on March 11, 2020. COVID-19 has since claimed millions of lives and has caused disasters.

Unfortunately, the symptoms of COVID-19 are very similar to those of coronavirus infection [1], that is, cough, runny nose, fever, headache, muscle aches, and diarrhea, which do not stand out from other respiratory tract infections, except for the high transmissibility and staggering case fatality rate (approximately 2%). Looking back to Severe Acute Respiratory Syndrome Coronavirus-1 (SARS-CoV-1), most infectious patients were febrile (98% percent from a previous report [2]). Additionally, symptoms other than classical upper respiratory tract infection may occur, such as smell and taste dysfunction [3, 4]. Moreover, some reports [2] revealed that some COVID-19 contraction might be due to asymptomatic or presymptomatic spread, which is quite a different scenario from the SARS pandemic in 2003 [5]. This also means that containing COVID-19 could be more difficult than SARS. Thus, implementing systematic screening for SARS-CoV-2 to identify potential COVID-19 cases might be a good shot to contain COVID-19, but this would raise the concern of the cost effectiveness as the reverse transcriptase-polymerase chain reaction (RT-PCR) tests might burn much Personal Protective Equipment (PPE) and cost health professional manpower as well. In contrast, overlooked COVID-19 cases may lead to further outbreaks as the transmission is exponentially fast. In this way, morbidities and mortalities could be even more disastrous. Moreover, economic costs are another issue and may result in further losses, such as mental breakdown and other sufferings.

Taipei City Hospital has seven branches, a public sector institute with 3,034 beds; the Ren-Ai Branch is a regional teaching hospital in urban Taipei with 655 beds. The Taipei City Hospital Ren-Ai Branch has also been responsible for caring for patients with mild and severe COVID-19 during the pandemic as well as ordinary healthcare since January 2020.

Taiwan has implemented proactive measures of border restrictions from China and other areas, as well as nonpharmaceutical interventions, including masking, washing hands, and social distancing. Together, Taiwan had kept COVID-19 at bay for more than 15 months until serial cases of local outbreaks occurred in May 2021. Since then, local cases have surged profoundly from 1000 cases to 14,503 local cases on August 4, 2021, and COVID-19 had claimed more than 700 lives just within these two months, and the case fatality rate was more than 5%.

Since the outbreak of COVID-19, the Taipei City Hospital Ren-Ai Branch has mandated the RT-PCR negative result for the patient within 72 hours before admission as a prerequisite to be admitted for preventing COVID-19 nosocomial infections. This study aimed to investigate whether this systematic screening test for COVID-19 effectively prevents COVID-19 nosocomial infections. Furthermore, asymptomatic ratios of COVID-19 were also investigated.

## 2. Materials and Methods

This was an observational cohort study. The number of SARS-CoV-2-positive tested patients was surveyed and retrieved from the Taiwan Center of Disease Control (CDC), with the most SARS-CoV-2 tests derived from the triage and test center. Categorical variables were summarized as counts and proportions and continuous variables as medians and interquartile ranges. Moreover, as background information, we demonstrated the outbreak surge and positive rate of RT-PCR tests during the same period from May 19 to July 27, 2021. The RT-PCR results of all patients with their caregivers admitted from May 15, 2021, to July 15, 2021, to the Taipei City Hospital Ren-Ai Branch, a regional teaching hospital in urban Taipei with more than 20,000 hospital admissions annually and relevant healthcare workers (HCWs), were included in this study. The infection control policy of Taipei City Hospital is strict. For example, the caregivers of admitted patients were restricted to only one member per patient based on the guidelines of Taiwan's Central Epidemic Command Center (CECC). Moreover, the patient, caregivers, and HCWs were required to undergo routine testing for SARS-CoV-2 (reverse transcriptase-polymerase chain reaction, RT-PCR); patients and caregivers required a negative RCR test result within 72 hours before admission and after that. Moreover, HCWs need to undergo PCR nasopharyngeal swab tests every week. Nasopharyngeal swabs were performed by a specially trained and dedicated team throughout the study period. An internally developed reverse transcription-quantitative nucleic acid assay was used to detect SARS-CoV-2 RNA.

**Figure 1:**
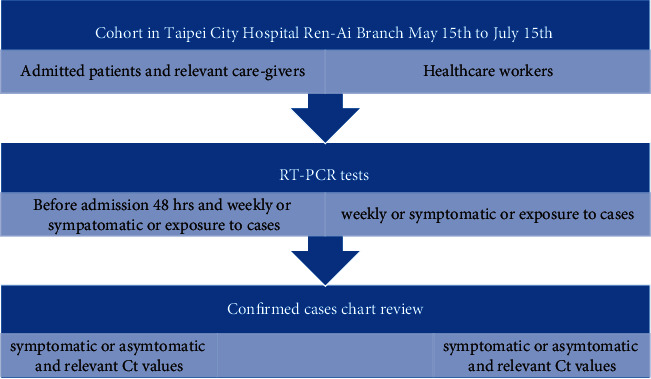
Schematic diagram of the research methodology.

Each lab-confirmed COVID-19 patient screened during the study period was retrospectively classified as symptomatic or asymptomatic for COVID-19 at the time of testing based on medical chart review. The classification criteria were the same as for the clinical consideration of COVID-19. They consistently applied during the screening period: shortness of breath and/or fever ≥38.0°C and/or new onset of anosmia or ageusia or dysgeusia, headache, sore throat, runny nose, cough, diarrhea, shortness of breath, and/or acute confusion or deterioration in the elderly unless otherwise explained. The percentage of positive SARS-CoV-2 and proportions of symptomatic and asymptomatic rates were calculated accordingly, and their relevant symptoms, as well as healthcare disciplines, were presented. Moreover, the healthcare professional infection rate was presented based on the same principles. SPSS Version 23 was used to perform descriptive statistics analyzing demographic characteristics and COVID-19 clinical presentations. Also, the schematic figure demonstrated our screening protocol (Figure 1).

## 3. Results

**Figure 2:**
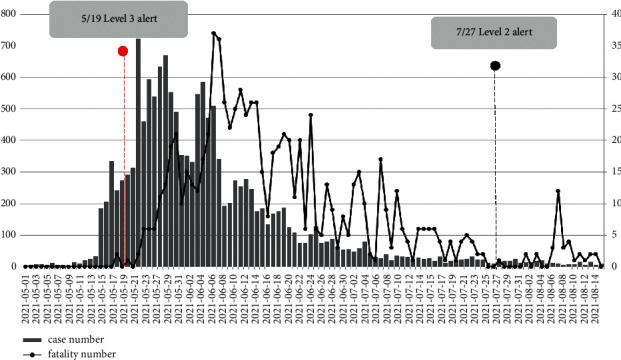
Trend of SARS-CoV-2 case numbers and fatality numbers in Taiwan.

The cases surged from May 19 to July 27, 2021; as shown in Figure 2, this local outbreak since May 2021 has made the CECC raise the national endemic alert tier (which includes four levels of alert) from level one to level two on May 11, 2021, and then to level three from May 15, to July 26, 2021. As the cases staggeringly increased, the case fatality rate increased as well, yet was slightly delayed temporarily. As of July 25, there were 14,262 local cases (versus 15,511 cases, including imported cases) and 778 fatalities in total. The prevalence of SARS-CoV-2 in Taiwan was 0.07%, and the case fatality rate was 5.03% at that time. This two-month period outbreak has been very dramatic for Taiwan, as the number of cases jumped from approximately 1000 to >14000, and mortality has increased from dozens to several hundred within a week's to a month's lag. Compared to the global trend, the prevalence in Taiwan is still very low, but our case fatality rate is extraordinarily high, around 5%, as compared to 2.15% globally.

### 3.1. Positive RT-PCR for the Admitted Group and HCWs

**Figure 3:**
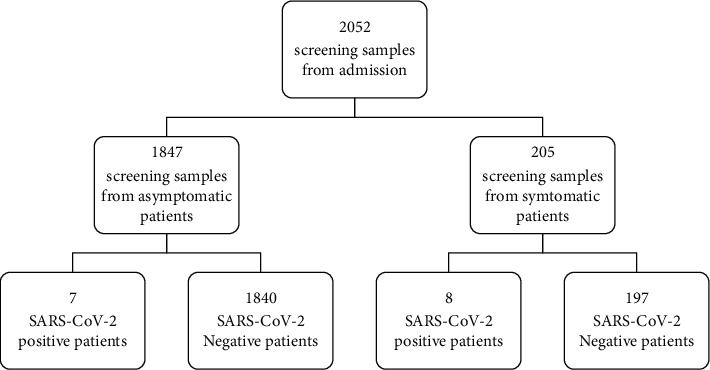
Calculus of SARS-CoV-2 case numbers (admission) in the Ren-Ai Branch of Taipei City Hospital from May 19, 2021, to July 27, 2021.

**Figure 4:**
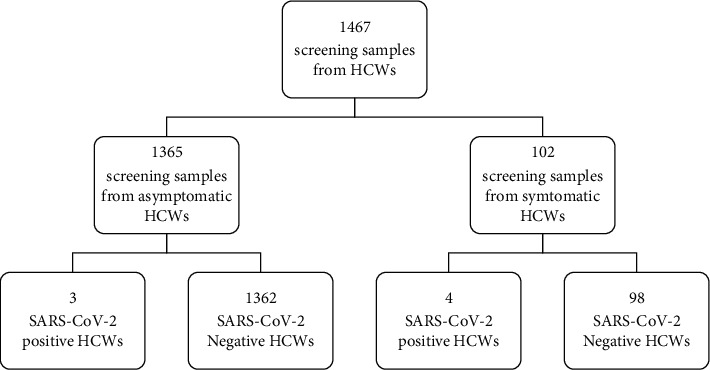
Calculus of SARS-CoV-2 case numbers (HCWs) in the Ren-Ai Branch of Taipei City Hospital from May 19, 2021, to July 27, 2021.

In total, there were 2,052 admitted patients and 1467 HCWs between May 19 and July 27, 2021, in Taipei City Hospital, Ren-Ai Branch. Among them, there were 15 positive cases in the admitted inpatient or caregiver group and seven positive cases in the HCW group. The positive rates were 15/2052 (0.73%) and 7/1467 (0.48%), respectively (Figures 3 and 4). Compared to the background positive rate of the RT-PCR test, it ranged from 8% to 0.02% in Taiwan. The odds ratios for the admission and HCWs were 10.67 and 18.53, respectively.

### 3.2. Relevant Symptoms of COVID-19-Positive Cases and Proportions of Asymptomatic COVID-19 Carriers

In the admission patient group (Table 1) (mean (SD) age, 46.1 [26.1] years; female (57.2%)), the symptoms and ratio in percentage were fever 33.3%, cough 20%, diarrhea 13.3%, sore throat 6.7%, abdominal pain 6.7%, and dyspnea 6.7%, The proportion asymptomatic COVID-19 carriers in the admitted patients group (caregivers) was 7/15 (46.6%). Among HCWs screened (population 1467, mean (SD) age, 42.6 (15.0) years; female (52.7%)), the symptoms and ratio in percentage were fever 42.9%, sore throat 28.6%, and cough 14.3%. The proportion of asymptomatic COVID-19 carriers in the HCW group was 3/7 (42.9%).

Soon after the outbreak in Taiwan, regular testing of HCWs was performed weekly, and only one out of seven positive cases had been vaccinated (first dose of AstraZeneca) one week before laboratory-confirmed diagnosis; the other six were not vaccinated.

Despite the results of the various cycle threshold (Ct) values in each laboratory-confirmed case, this study revealed that a low Ct value did not necessarily correlate with a higher rate of symptomatic presentation.

## 4. Discussion

The WHO has stated that the mortality rate of COVID-19 is approximately 2% [6], indicating that, similar to influenza, many infected people may overcome the disease without needing hospitalized treatment for severe illness or ICU admission. Given the novelty of the virus, the lack of immunity has resulted in a high likelihood of people around the world becoming ill upon the first contact with SARS-CoV-2. Under a pandemic scenario, the management approach of asymptomatic carriers who may spread the virus is one challenge that needs to be mitigated.

Asymptomatic carriers are likely to spread the virus. According to an article published in the New England Journal of Medicine (NEJM) on the evacuation of German nationals from Wuhan, it can be concluded that transmission of the virus may occur even in SARS-CoV-2 carriers without fever or other symptoms of infection [7]. According to the study, the primary target or receptor for SARS-CoV-2 is angiotensin-converting enzyme 2 (ACE2) in type II pneumocyte in the lungs, goblet cells, and epithelial cells in the nose, which causes postinflammatory immunoreaction, so the severity of viral infection is closely related to the maturity and binding capacity of ACE2. Therefore, we propose that the lower level and insufficient binding capacity of ACE2 is a biological factor for asymptomatic or mild symptomatic clinical presentation [7, 8].

SARS-CoV-2 is primarily transmitted through droplets and close contact with the elderly and people with chronic diseases, which are considered high-risk groups [9]. The vulnerability of the elderly is due to age-related decline and dysregulation of immune function, that is, immunosenescence and inflammaging [10]. As mentioned above, from the outbreak and rapid spread of COVID-19 in the long-term care facilities in King County, Washington State, 23 residents (30%) tested positive, and among them, 10 (43%) presented with symptoms on the day of testing, while 13 (57%) did not [11]. Therefore, symptom-based screening may fail to identify nearly half of the people infected with COVID-19.

Besides the study of the elderly, clinical data of children and infants infected with COVID-19 have also been evaluated. According to the results of a study from Wuhan [12], 15.8% (27/171) of infected children under 15 years of age were asymptomatic, and the incidence was lower than that of the whole population, which may be due to a special immune response and lower levels of ACE2 [8, 12]. In a case report [13], the stool samples of an asymptomatic child were tested positive by PCR 17 days after the last exposure to the virus and remained positive for at least another nine days. However, PCR tests on respiratory tract samples of the child were negative.

A previous study [14] examined 26 confirmed asymptomatic carriers. The results showed that the changes in blood biochemical and inflammatory indexes were very small, as were the changes observed through chest Computed Tomography (CT) examinations. Concerning chest CT, a retrospective study [15] on its association with the clinical course of asymptomatic carriers found that, among the 58 asymptomatic cases with initial normal laboratory test results, the main CT features were ground-glass opacities (GGOs) (55 cases, 95%). Later, 16 patients (28%) showed decreased lymphocyte count and increased C-reactive protein (CRP) levels and experienced symptoms primarily including fever, cough, and fatigue. In summary, it can be deduced that CT imaging of asymptomatic carriers may have distinct characteristics. Since asymptomatic carriers are known as the “hidden disseminators,” attention should be paid to tracking and monitoring.

In another report [16], 55 asymptomatic carriers were investigated by PCR testing of throat swab samples, and all of them were confirmed positive for COVID-19 by laboratory tests. Research evidence has suggested that middle-aged individuals who have been in close contact with infected people are more likely to be asymptomatic carriers, and most of them would progress to mild and moderate pneumonia during hospitalization.

Among Orthocoronavirinae, for SARS, in a study for HCWs in Singapore in 2005, only 13.3% of the infected were asymptomatic, and asymptomatic SARS was associated with lower SARS antibody titers and higher use of masks [17]. In a review of 10 studies [18], the asymptomatic rate of Middle-East Respiratory Syndrome (MERS-CoV) was 12.5% to 91.7%, and asymptomatic individuals were less likely to have underlying conditions (42%) compared to 86% of fatal cases. According to a review of six studies, asymptomatic SARS-CoV-2 accounts for 1.6% to 56.5% of infected patients [8].

### 4.1. Positive Rate of PCR Tests in This Systematic Screening

The positive rates were 0.73% (15/2052) and 0.48% (7/1467) for admitted patients and HCWs, respectively. Compared to the background positive rate of the RT-PCR test, it has been reported to range from 8% to 0.02% in Taiwan. Furthermore, if the PCR test positive rate is higher than 5%, it implies that the testing capacity is insufficient, which also mirrored the ramping up of test capacities as mentioned above; that is, through the expanding, PCR test capacities helped to control this astonishing local outbreak.

### 4.2. Asymptomatic Proportion of COVID-19 and the Presenting Symptoms

In this study, the asymptomatic proportion was 7/15 (46.6%) in the group of admitted patients and 3/7 (42.9%) in the HCW group. This strenuous testing with high economic costs paid off. As the asymptomatic proportion was unexpectedly high, if precautions were not that drastic, the spread of COVID-19 nosocomial infection could have cost even higher prices.

Conversely, the presenting symptoms of COVID-19 in these dozen cases were not peculiar; most presented with symptoms of fever (33.3%), cough (20%), diarrhea (13.3%), sore throat (6.7%), abdominal pain (6.7%), and dyspnea (6.7%). These symptoms were not distinct from those of a common upper respiratory tract infection (Table 1).

Universal screening of admitted patients may be crucial and necessary, especially in asymptomatic patients. Additionally, regular screening for healthcare providers is important during the pandemic. In conclusion, systemic PCR testing for admitted patients and healthcare providers during the pandemic is recommended.

### 4.3. Strength and Limitation

The strength of this study is that it was an observational retrospective cohort study, which might reflect real-world evidence, as the pandemic still overwhelms global citizens, providing evidence of the significance of universal testing to contain COVID-19. The limitation of this study is that it was not a prospective study, and the results may not be objective or biased, and the observational period was quite short as it was two months long.

## 5. Conclusions

According to the literature in various countries, the estimated number of asymptomatic carriers of COVID-19 varies globally [8]. In the early days of the pandemic, asymptomatic carriers accounted for 1-2% of cases [19]. In contrast, recent studies in Europe and America have demonstrated that this proportion seems to be far more than imagined. Consolidated studies from the scientific journal Nature highlighted that mildly ill or asymptomatic patients might account for 60% of all infected cases [20]. Moreover, the transmissibility of asymptomatic carriers may be higher than expected. Although the proportion of asymptomatic carriers is important, the transmissibility of asymptomatic carriers is more concerning. The WHO has not yet conducted any large-scale or in-depth research on asymptomatic carriers and even emphasized early on during the epidemic that such population groups were extremely rare. As mentioned above, increased studies have revealed that asymptomatic carriers substantially impact the epidemic and that asymptomatic transmission is very common. Therefore, the threat of asymptomatic transmission to the spread of the pandemic should not be neglected.

This local outbreak of Taiwan, especially in Taipei City, is unprecedented and paramount and has claimed hundreds of lives, tens of thousands of cases, and enormous economic costs. As care providers and gatekeepers of infectious diseases, Taipei City Hospital has to perform regular PCR results of admitted patients and HCWs to achieve these goals. The results revealed a low positive rate of less than 1%, but the asymptomatic proportions ranged from 42% to 46%. The implications of such a high proportion of asymptomatic proportions referred to the difficulties in containing SARS-CoV-2, and so, we did not perform systematic screening PCR tests even at the expense of high economic costs. On the contrary, through this universal screening of SARS-CoV-2, PCR tests helped contain the spread of COVID-19. In this way, it helped protect HCWs and patients.

In this study, the results revealed a low positive rate of less than 1%, but the asymptomatic proportions could range from 42% to 46%, which bolsters that systematic screening effectively controlled COVID-19 and might be an exemplar to other similar scenarios. Universal screening of admitted patients may be important and necessary, especially in asymptomatic patients. In addition, regular screening for healthcare providers is also important during this pandemic, and it is recommended that admitted patients and healthcare providers undergo systemic PCR testing.

## Figures and Tables

**Table 1:** Demographic data of SARS-CoV-2-positive admission patients and HCWs of systematic screening.

	Admission patients	HCWs
	*N* (%)	*N* (%)
Age (mean ± SD)	46.1 ± 26.1	42.6 ± 15.0
Gender		
M	7 (47%)	3 (43%)
F	8 (53%)	4 (57%)
Asymptomatic	7 (47%)	3 (43%)
Symptom	8	4
Fever	5	4
Cough	3	1
Diarrhea	2	
Sore throat	1	2
Abdominal pain	1	
Dyspnea	1	

## Data Availability

The authors declare that all data supporting the findings of this study are available within the article.
